# Milk fat globule membrane supplementation to obese rats during pregnancy and lactation promotes neurodevelopment in offspring *via* modulating gut microbiota

**DOI:** 10.3389/fnut.2022.945052

**Published:** 2022-08-15

**Authors:** Qichen Yuan, Han Gong, Min Du, Tiange Li, Xueying Mao

**Affiliations:** ^1^Key Laboratory of Functional Dairy, College of Food Science and Nutritional Engineering, Ministry of Education, China Agricultural University, Beijing, China; ^2^Department of Animal Sciences, Washington State University, Pullman, WA, United States; ^3^Henan Engineering Technology Research Center of Food Processing and Circulation Safety Control, College of Food Science and Technology, Henan Agricultural University, Zhengzhou, China

**Keywords:** milk fat globule membrane, maternal high-fat diet, neurodevelopment, gut microbiota, inflammation

## Abstract

Pre-pregnancy obesity and high-fat diet (HFD) during pregnancy and lactation are associated with neurodevelopmental delay in offspring. This study aimed to investigate whether milk fat globule membrane (MFGM) supplementation in obese dams could promote neurodevelopment in offspring. Obese female rats induced by HFD were supplemented with MFGM during pregnancy and lactation. Maternal HFD exposure significantly delayed the maturation of neurological reflexes and inhibited neurogenesis in offspring, which were significantly recovered by maternal MFGM supplementation. Gut microbiota analysis revealed that MFGM supplementation modulated the diversity and composition of gut microbiota in offspring. The abundance of pro-inflammatory bacteria such as *Escherichia shigella* and *Enterococcus* were down-regulated, and the abundance of bacteria with anti-inflammatory and anti-obesity functions, such as *Akkermansia* and *Lactobacillus* were up-regulated. Furthermore, MFGM alleviated neuroinflammation by decreasing the levels of lipopolysaccharides (LPS) and pro-inflammatory cytokines in the circulation and brain, as well as inhibiting the activation of microglia. Spearman’s correlation analysis suggested that there existed a correlation between gut microbiota and inflammation-related indexes. In conclusion, maternal MFGM supplementation promotes neurodevelopment partly *via* modulating gut microbiota in offspring.

## Introduction

Maternal nutrition during the early life affects fetal growth and development, which programs the health status of offspring in later life (1). Maternal obesity and high-fat diet (HFD) intake increase the risk of metabolic syndromes such as obesity, insulin resistance, and hyperlipidemia in adulthood (2, 3). Maternal HFD from pre-pregnancy to lactation affects the neurodevelopment of offspring and exacerbates the occurrence of behavioral and emotional disorders (4).

The hippocampus is a key brain region responsible for learning and memory (5). The dentate gyrus (DG) of the hippocampus has a unique neurogenesis capacity, which generates new neurons *via* migration, proliferation and differentiation. Alterations in neurogenesis are associated with neurodevelopmental abnormalities and neurological disorders (6). The long-term and complex features of neurodevelopment make it highly sensitive to environmental factors, especially nutritional status in early life. Epidemiological studies revealed that pre-pregnancy overweight and obesity were correlated with lower scores for verbal recognition in children. Prenatal exposure to maternal obesity leads to lower intelligence quotients, delayed mental development and increased emotional and behavior problems (7–9). The maturation of neurological reflex behaviors such as free-fall righting and negative geotaxis in offspring were delayed by maternal HFD during lactation (10). Offspring of HFD dams were born obese, and presented impaired DG neurogenesis and hippocampal-dependent spatial cognitive function (6, 11). These studies show that maternal obesity and HFD could adversely affect the neurodevelopment of offspring.

Neurodevelopment can be substantially affected by the gut microbiota. Hippocampal neurogenesis was impaired at weaning in germ-free mice compared with normal mice, suggesting that neurogenesis could be regulated by gut microbiota (12). In the absence of gut microbiota, the main neuroimmune cells microglia were stunted and subsequently remained immature with limited immune responses to viruses and infections (13). Therefore, disturbance of the gut microbiota during development may affect neurodevelopment and adversely affect brain health in later life (14). The gut microbiota of infants is highly sensitive to disturbance of environmental factors such as dietary changes. In cohort studies, the diversity of gut microbiota altered and the abundance of *Bifidobacterium* and *Bacteroides* were decreased in the offspring exposed to maternal obesity or HFD, which had negative effects on energy acquisition and early immune development (15–17). In rodents, maternal HFD before and during pregnancy impaired the gut microbiota of both dams and offspring (18, 19). The α-diversity tended to decrease and the *Firmicutes*/*Bacteroidetes* ratio increased significantly in the gut microbiota of 2-week-old offspring from HFD dams (19). Maternal diet may affect the behavior of offspring *via* altering the gut microbiota. It was found that offspring exposed to maternal HFD exhibited severe social deficits, which was associated with changes in gut microbiota. However, postnatal supplementation with *Lactobacillus reuteri* (depleted due to maternal HFD) in offspring improved social behavior, indicating a causal link among maternal diet, gut microbiota, and neurodevelopment (20). Therefore, modulating the gut microbiota of offspring by intervention of maternal nutrition is an effective way to affect neurodevelopment of offspring.

Milk fat globule membrane (MFGM) composes of a three-layer membrane surrounding lipid droplets in milk, rich in glycoproteins and polar lipids. MFGM has shown the function of promoting infant growth and development, regulating immunity, and improving glycolipid metabolism. In a prospective, double-blind and randomized controlled trial, supplementation with MFGM narrowed the gap in neurodevelopment between infant formula-fed and breast-fed infants (21). Growth-restricted suckling mice supplemented with MFGM from birth to weaning increased cognitive scores (22). In our previous study, supplementation of milk polar lipids in obese dams enhanced offspring neurodevelopment *via* suppressing brain insulin resistance (23). These studies suggested that MFGM supplementation during the early life was beneficial to neurodevelopment. In addition, MFGM could alleviate endotoxemia by improving the gut microbiota of obese mice (24). MFGM supplementation during pregnancy and lactation ameliorated dysbiosis of obese rat dams (25), indicating that MFGM is favorable for obesity-related gut microbiota and inflammatory status. Based on these studies, the effects of MFGM supplementation to HFD-induced obese dams during pregnancy and lactation on the neurodevelopment of offspring were measured, and the corresponding changes in the gut microbiota and inflammatory responses were explored, which promoted neurodevelopment.

## Materials and methods

### Animals

Three to four-week-old female Sprague-Dawley rats were purchased from Beijing Vital River Laboratory Animal Technology Company Limited (Beijing, China), and were housed in the animal room of China Agricultural University under the environment of 22 ± 1°C with 12 h light-12 h dark cycle. After 1 week of acclimation, the rats were randomly divided into two groups: the control group was fed a control diet (*n* = 12) (10% calories from fat, D12450J, Research Diets), and the HFD group was fed a HFD (*n* = 12) (60% calories from fat, D12492, Research Diets) for 8 weeks. Body weight was weighed every week. Then female rats were caged with 10-week-old male rats at a ratio of 2:1 at 8:00 p.m. with free access to food and water, and the vaginal smears was examined at 8:00 a.m. the next day. Once the vaginal smears were found, it was recorded as the first day of pregnancy. After mating, the control group of pregnant mice were randomly divided into 2 groups: one group was fed normal diet (CON, *n* = 6), and the other group was fed normal diet supplemented with 400 mg/kg BW MFGM (CON + MFGM, *n* = 6). Similarly, pregnant rats in the HFD group were randomly divided into 2 groups: one group was fed HFD (45% calories from fat, D12451, Research Diets) (HFD, *n* = 6), and the other group was fed with HFD supplemented with 400 mg/kg BW MFGM (HFD + MFGM, *n* = 6). The caloric information of diets was shown in Supplementary Table 1. MFGM was provided by Arla Co. (Sønderhøj, Viby J, Denmark). All groups maintained on the above diets until the end of lactation. At birth, pups were weighed and sex-determined, and litters were culled to 8 pups. Offspring were kept with their dams until postnatal day (PND) 21 (weaning). The animal study was approved by the Ethics Committee of China Agricultural University (License No. KY. 180026).

### Reflex development

#### Righting reflex

Righting reflex was performed as previously described (26). On PND 3-7, the pups were placed on their backs on a rough wooden board. The day when pups turned over from the supine position to prone position within 5 s was recorded.

#### Cliff avoidance

Cliff avoidance was performed as previously reported (26). On PND 5-8, the pups were placed on the edge of a suspended plate with their nose and front paws over the edge. The day pups withdrew their nose and forepaws from the edge within 10 s was recorded.

#### Negative geotaxis

Negative geotaxis was performed as previously described (27). On PND 6-11, the pups were placed head down on a flat and rough wooden board with an inclination angle of 45°. The day the pups turned around and climbed up the board within 20 s was recorded.

### Tissues collection and blood sampling

For offspring on PND 21, rats were fasted overnight and anesthetized with ethyl ether, and the trunk blood of the decapitated rats was rapidly collected. Blood samples were centrifuged at 1,000 g for 20 min at 4°C to obtain serum. Serum was stored at −80^°^C until needed. For immunohistochemical analysis, the brain was rapidly removed and fixed in 4% PFA. For western blot analysis, the brain was immediately frozen in liquid nitrogen, and stored at −80^°^C until needed.

### Immunohistochemical examination

Immunohistochemical examination was performed as previously described (23). Coronal brain sections were sliced using a freezing microtome (Leica, Germany) and pretreated with 3% hydrogen peroxide to block endogenous peroxidase activity. The sections were incubated with 3% bovine serum albumin in PBS and were incubated overnight at 4^°^C with primary antibodies anti-doublecortin (Abcam, ab18723) and anti-Ki67 (Abcam, ab16667). After washing in PBS for three times, sections were incubated with a biotinylated secondary antibody (Abcam, ab205718) for 2 h at room temperature. Following another wash with PBS, sections were subjected to 3,3’-diaminobenzidine (DAB). Sections were counterstained with hematoxylin, dehydrated, and cleared in xylene. The sections were observed under the Olympus IX 73 microscope (Olympus Corporation Tokyo, Japan) and the average optical density were quantified using Image-Pro Plus 6.0 software.

### 16S rRNA gene sequence analysis

Fresh feces from weaned offspring were collected in dry sterile centrifuge tubes, and stored at −80°C. 16S rDNA high-throughput sequencing was conducted by Majorbio BioPharm Technology Co., Ltd. (Shanghai, China). Samples were thawed on ice, and total DNA was extracted with an E.Z.N.A. soil DNA kit (Omega Bio-Tek, Norcross, GA, United States). The DNA quality was detected by 1% agarose gel electrophoresis. The V3-V4 regions of the bacterial 16S rRNA gene was amplified with primers 338F (5’-ACTCCTACGGGAGGCAGCAG-3’) and 806 R (5’-GGACTACHVGGGTWTCTAAT-3’). PCR reactions were performed under the following program: pre-denaturation at 95°C for 3 min, 30 cycles (denaturation at 95°C for 30 s, annealing at 55°C for 30 s, extension at 72°C for 45 s), extension at 72°C for 10 min, and then maintained at 10°C until use. The PCR products were detected by 2% agarose gel electrophoresis, and the purified and amplified fragments were used to construct a PE 2*300 library. After the library was verified, the Illumina MiSeq PE300 platform was used for sequencing according to the standard procedure. Raw fastq files were filtered and quality controlled by Trimmomatic, and spliced by FLASH. The sequences were clustered into OTUs according to the similarity of 97%, and the OTUs sequences were compared with the Silva database using RDP Classifier algorithm to analyze the taxonomic classification of gut microbiota.

### Inflammatory factors analysis

Levels of LPS, IL-1β, IL-6, and TNF-α in the serum and brain were determined using ELISA assay kits according to the manufacturer’s instructions (Cusabio Life science, Wuhan, Hubei, China).

### Western blot

Western blot was performed as previously described (28). Brains were weighed and homogenized in ice cold RIPA buffer (Beyotime, Shanghai, China) containing 1% protease phosphatase inhibitor (Beyotime, Shanghai, China), followed by centrifugation at 10,000 g for 15 min at 4°C to collect the supernatant. The protein concentration was determined by the BCA protein assay reagent (Tiangen Biotech, Beijing, China). The samples were stored at −20°C. After separation by 10% sodium dodecyl sulfate polyacrylamide gel electrophoresis (SDS-PAGE), the proteins were transferred to activated polyvinylidene difluoride (PVDF) membranes (Millipore, Bedford, MA, United States). Membranes were blocked in 5% skim milk for 1 h at room temperature, and then incubated with primary antibodies targeting Iba1 (Abcam, ab178846, 1:1,000) and β-actin (Bioss, #bs-0061R, 1:1,000) overnight at 4°C. After being washed with TBST solution for 5 times, membranes were incubated with the horseradish-peroxidase-conjugated secondary antibody for 1 h at room temperature. The bands were visualized by enhanced chemiluminescence reagent (Millipore, Bedford, MA, United States) and quantified using ImageJ software.

### Statistical analysis

Values are expressed as means ± standard error of the mean (SEM). Significant differences were determined using SPSS software (version 23.0, IBM Corp., United States). One-way analysis of variance (ANOVA) followed by *post hoc* Tukey’s multiple comparison test was used for parametric analysis of variance between groups, and Student’s *t*-test was used for comparing two groups. Mann-Whitney non-parametric tests were performed to determine the differences in reflex experiments. Significance was set at *P* < 0.05.

## Results

### Maternal milk fat globule membrane supplementation decreased body weight and promoted neurobehavioral development in high-fat diet offspring

**FIGURE 1:**
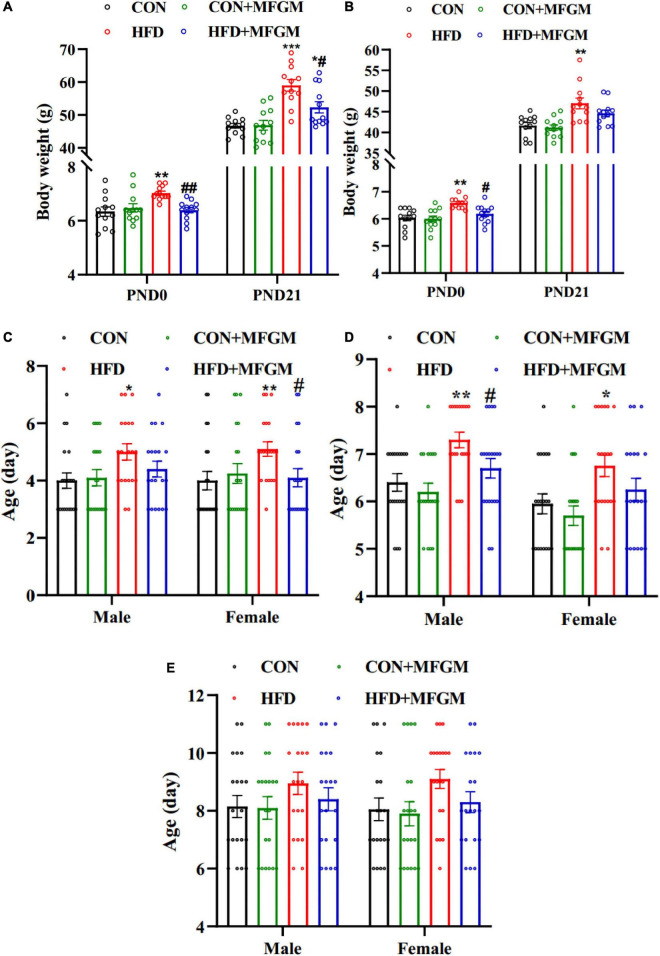
Maternal supplementation with milk fat globule membrane during pregnancy and lactation promoted neurobehavioral development in offspring from HFD dams before weaning. **(A)** Body weight of male offspring. **(B)** Body weight of female offspring, *n* = 12. The average age of reaching the criterion of right reflex **(C)** cliff avoidance **(D)** and negative geotaxis **(E)**, *n* = 20. Values are mean ± SEM. *P < 0.05 vs. CON group. ^#^*P* < 0.05 vs. HFD group. **P < 0.01 vs. CON group. ^##^*P* < 0.01 vs. HFD group. ***P < 0.001 vs. CON group.

Food intake of dams during pregnancy and lactation was shown in Supplementary Figure 1, and MFGM supplementation didn’t influence food intake of HFD dams. The body weight of the offspring at birth and weaning was measured. As shown in Figure 1A, maternal HFD significantly increased the body weight of male offspring compared with that of CON offspring on PND 0 and 21. Maternal MFGM supplementation substantially decreased the body weight of offspring. Similar results were observed in the body weight of female offspring (Figure 1B). The body weight of female offspring born from HFD dams was higher than that of the CON offspring on PND 0 and 21 (*P* < 0.01), which was suppressed due to MFGM intervention in obese dams.

To evaluate the effect of MFGM on the development of neurological reflexes in offspring, righting reflex, cliff avoidance and negative geotaxis were performed. As shown in Figures 1C,D, there was no significant difference in the average age of finishing reflexes of the male and female offspring between the CON group and the CON + MFGM group, while HFD offspring had a significantly delayed time to finish righting reflex and cliff avoidance. Maternal MFGM intervention significantly promoted righting reflex development in female offspring, meanwhile there was no significant difference between the HFD + MFGM group and the CON group in male offspring. Besides, cliff avoidance occurred obviously earlier in HFD + MFGM male offspring than that in HFD male offspring, while no significant difference was observed in female offspring between HFD + MFGM group and CON group. The average age of finishing negative geotaxis displayed no differences among the four groups (Figure 1E). These results indicated that MFGM supplementation in obese dams during pregnancy and lactation could promote neurobehavioral development in offspring.

### Maternal milk fat globule membrane supplementation promoted neurogenesis in high-fat diet offspring

**FIGURE 2:**
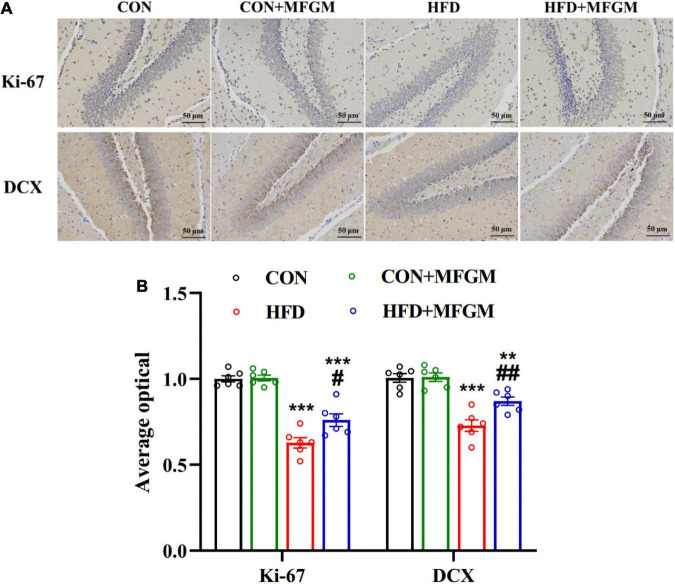
Maternal supplementation with milk fat globule membrane during pregnancy and lactation promoted neurogenesis in weaned offspring from HFD dams. **(A)** Immunohistochemical staining and average optical density value of Ki-67 and DCX in the dentate gyrus of the offspring. **(B)** Average optical density value of Ki-67 and DCX, *n* = 6. Values are mean ± SEM. ^#^*P* < 0.05 vs. HFD group. **P < 0.01 vs. CON group. ^##^*P* < 0.01 vs. HFD group. ***P < 0.001 vs. CON group.

To evaluate the effect of maternal MFGM supplementation on the hippocampal neurogenesis of the offspring at weaning, the positive cells of Ki-67, a marker of cell proliferation and doublecortin (DCX), a marker of newborn neurons, were determined. As shown in Figure 2, compared with the CON group, the optical density of hippocampal Ki-67 in the offspring of the HFD group was dramatically decreased (*P* < 0.001). MFGM supplementation in obese dams notably recovered the expression of Ki-67 (*P* < 0.05). Similarly, the optical density of the hippocampal DCX of the HFD offspring was notably decreased compared with the CON group, while this reduction was markedly prevented by maternal MFGM supplementation (*P* < 0.01). These results suggest that maternal MFGM supplementation attenuated the impairment of neurogenesis in weaned offspring from obese dams.

### Maternal milk fat globule membrane supplementation modulated the diversity of gut microbiota in high-fat diet offspring

**FIGURE 3:**
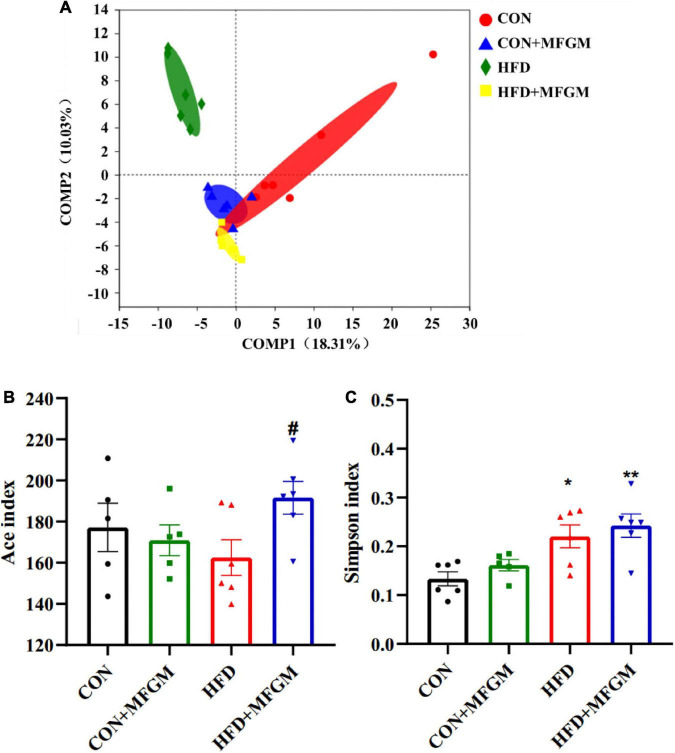
Maternal supplementation with milk fat globule membrane during pregnancy and lactation modulated the overall structure of gut microbiota in weaned offspring from HFD dams. **(A)** Principal component analysis plot of the gut microbiota at the OTU level. The α-diversity of gut microbiota depicted according to Ace index **(B)** and Simpson index **(C)** at the OTU level. Values are mean ± SEM, *n* = 5–6. *P < 0.05 vs. CON group. ^#^*P* < 0.05 vs. HFD group. ^##^*P* < 0.01 vs. HFD group.

According to the PLS-DA (Figure 3A), there was a clear separation between the CON group and the HFD group, indicating that maternal HFD induced differences in the bacterial community structure of weaned offspring. Reduced gap and an overlap between HFD + MFGM and CON groups was observed, demonstrating that maternal MFGM supplementation restored the gut microbiota structure of offspring. The biological diversity within the sample was reflected by α-diversity, and the Ace index and the Simpson index were used to evaluate the community richness and diversity of the gut microbiota. Compared with the CON offspring, the Ace index of the gut microbiota in the HFD offspring did not change significantly but had a lower value, while HFD + MFGM offspring presented an increase in the Ace index (*P* < 0.05) (Figure 3B). The Simpson index in the HFD offspring was higher than that in the CON offspring (*P* < 0.01) (Figure 3C), indicating that maternal HFD reduced the species diversity of gut microbiota in weaned offspring. MFGM supplementation to HFD dams did not significantly change the Simpson index in offspring. These results indicate that maternal MFGM administration modulated the diversity of gut microbiota in HFD offspring.

### Maternal milk fat globule membrane supplementation modulated the composition of gut microbiota in high-fat diet offspring

**FIGURE 4:**
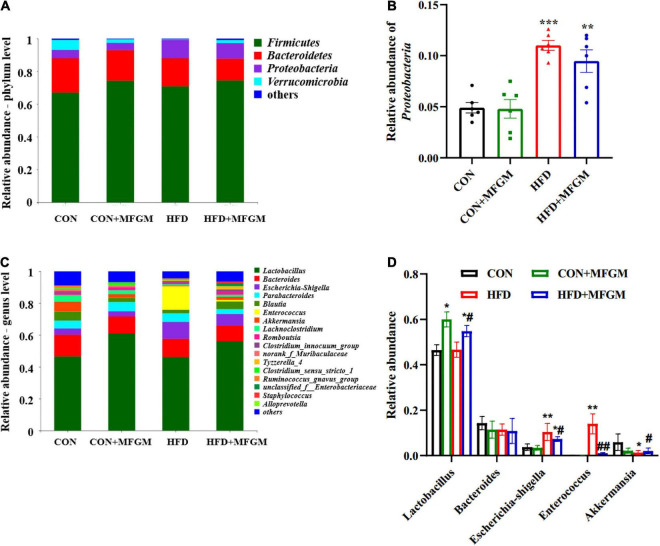
Maternal supplementation with milk fat globule membrane during pregnancy and lactation regulated the composition of gut microbiota in weaned offspring from HFD dams. **(A)** Relative abundance of gut microbiota at the phylum level. **(B)** Relative abundance of *Proteobacteria*. **(C,D)** Relative abundance of gut microbiota at the genus level. Values are mean ± SEM, *n* = 5–6. *P < 0.05 vs. CON group. ^#^*P* < 0.05 vs. HFD group. **P < 0.01 vs. CON group. ^##^*P* < 0.01 vs. HFD group. ***P < 0.001 vs. CON group.

As shown in Figure 4A, *Firmicutes*, *Bacteroidetes*, *Proteobacteria*, and *Verrucomicrobia* were the main dominant phyla in the gut microbiota of weaned offspring at the phylum level. Compared with the CON group, the relative abundance of *Proteobacteria* increased significantly in the HFD group (*P* < 0.001), while the HFD + MFGM offspring presented lower abundance of *Proteobacteria* (Figure 4B). At the genus level (Figures 4C,D), compared with the CON group, the relative abundance of *Lactobacillus* in the CON + MFGM group was significantly increased (*P* < 0.05). The relative abundance of *Lactobacillus* was also higher in the HFD + MFGM offspring compared with HFD offspring (*P* < 0.05). Meanwhile, the relative abundance of *Akkermansia* was reduced by 78% in HFD offspring compared with CON offspring, while HFD + MFGM offspring presented an increase in the relative abundance of *Akkermansia*. Compared with the CON group, the relative abundance of *Escherichia shigella* and *Enterococcus* in the HFD offspring were upregulated by 181 and 154%, respectively, which was recovered by MFGM supplementation. These results suggest that maternal MFGM administration improved the composition of gut microbiota in offspring born to HFD dams.

### Maternal milk fat globule membrane supplementation decreased serum pro-inflammatory factors in high-fat diet offspring

To determine whether the effects of MFGM on the gut microbiota could improve inflammation in HFD offspring, the levels of serum pro-inflammatory factors of the weaned offspring were measured firstly. As shown in Table 1, compared with the CON group, the serum levels of IL-1β, IL-6, TNF-α, and LPS were up-regulated by 50, 33, 11, and 46%, respectively, in HFD offspring, which were markedly reversed by MFGM intervention.

**TABLE 1:** Maternal supplementation with milk fat globule membrane during pregnancy and lactation alleviated serum levels of inflammatory factors in weaned offspring from HFD dams.

	IL-1β (pg/mL)	IL-6 (pg/mL)	TNF-α (pg/mL)	LPS (EU/L)
CON	15.64 ± 1.54	79.86 ± 3.72	86.42 ± 3.62	355.45 ± 18.98
CON + MFGM	15.82 ± 1.70	82.33 ± 3.19	87.92 ± 2.27	328.69 ± 30.32
HFD	31.15 ± 0.95***	106.51 ± 2.31***	95.73 ± 2.84**	519.09 ± 40.99***
HFD + MFGM	23.06 ± 1.27***^###^	86.99 ± 3.58^###^	90.52 ± 1.55^#^	422.63 ± 12.89***^##^

Values are mean ± SEM, n = 6.

#P < 0.05 vs. HFD group. **P < 0.01 vs. CON group. ^##^*P* < 0.01 vs. HFD group. ***P < 0.001 vs. CON group. ^###^*P* < 0.001 vs. HFD group.

### Correlation between gut microbiota and serum pro-inflammatory factors

**FIGURE 5:**
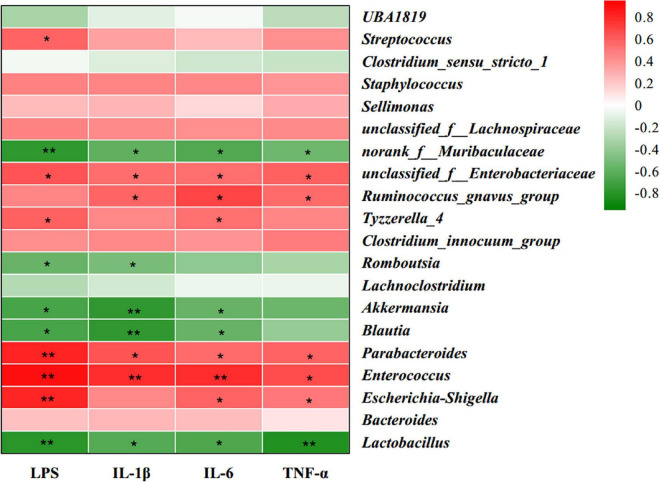
Heatmap of Spearman’s correlation between gut microbiota (the 30 most abundant species at the genus level) and serum inflammatory factors. Red color represents a positive correlation, while green color represents a negative correlation. *n* = 6. *0.01 < *P* ≤ 0.05, **0.001 < *P* ≤ 0.01.

To examine the correlation between gut microbiota and inflammatory responses, Spearman’s correlation analysis was used to calculate the correlations of the top 30 most abundant bacteria at the genus level with serum inflammation-related parameters (Figure 5). The heatmap reflected significant negative correlations between serum pro-inflammatory factors (LPS, IL-1β, IL-6, and TNF-α) and *Lactobacillus*, *Blautia*, *Akkermansia*, and *norank_f_Muribaculaceae*, indicating that these bacteria may be beneficial for the alleviation of inflammatory response in offspring. Serum pro-inflammatory factors were positively correlated with *Escherichia-Shigella*, *Enterococcus*, *Parabacteroides*, *Tyzzerella_4*, *Ruminococcus_gnavus_group*, and *unclassified_f_Enterobacteriaceae*, indicating that these bacteria may be involved in the inflammatory state of HFD offspring.

### Maternal milk fat globule membrane supplementation alleviated neuroinflammation in high-fat diet offspring

**FIGURE 6:**
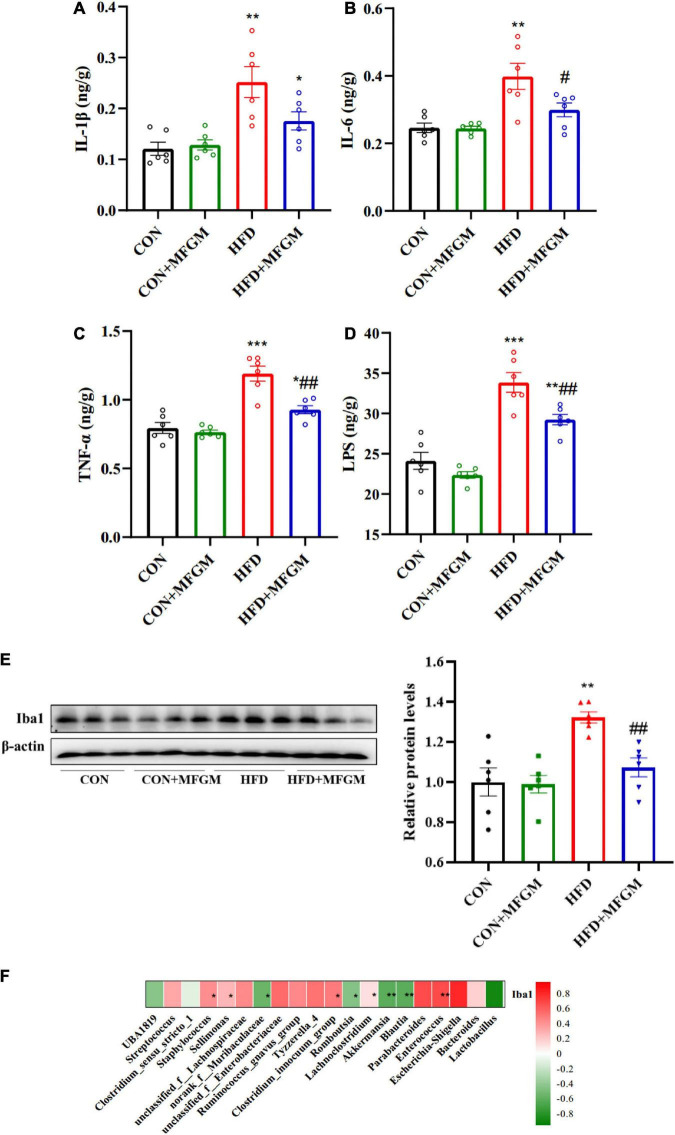
Maternal supplementation with milk fat globule membrane during pregnancy and lactation alleviated neuroinflammation in weaned offspring from HFD dams. IL-1β **(A)**, IL-6 **(B)**, TNF-α **(C)** and LPS **(D)** levels in the brain. **(E)** Representative image and relative quantitative analysis of Iba1 in the brain by Western blot. **(F)** Heatmap of Spearman’s correlation between gut microbiota (the 30 most abundant species at the genus level) and Iba1. Values are mean ± SEM, *n* = 6. *P < 0.05 vs. CON group. ^#^*P* < 0.05 vs. HFD group. **P < 0.01 vs. CON group. ^##^*P* < 0.01 vs. HFD group. ***P < 0.001 vs. CON group.

Circulating inflammatory factors could impair and cross the blood-brain barrier to induce neuroinflammation. To further investigate the effects of MFGM on the neuroinflammation in HFD offspring, the levels of brain pro-inflammatory factors and the protein expression of microglia marker Iba1 were analyzed. As shown in Figures 6A–D, compared with the CON offspring, the brain levels of IL-1β, IL-6, TNF-α, and LPS were notably increased in HFD offspring. The brain levels of IL-6, TNF-α and LPS were obviously down-regulated by MFGM intervention, and the value of IL-1β was lower than HFD group in HFD + MFGM group, suggesting that MFGM administration to HFD dams could attenuate cerebral inflammatory response. Furthermore, microglia activation is a marker of neuroinflammation. In order to explore the effects of maternal MFGM supplementation on microglia activation of weaned offspring, the protein expression of Iba1 was measured (Figure 6E). Compared with the CON group, the protein content of Iba1 in the brain of HFD offspring was significantly increased, which was recovered by maternal MFGM intervention. These data showed that MFGM can alleviate neuroinflammation in HFD offspring. The correlation analysis identified significant negative correlations between *Lactobacillus*, *Blautia*, *Akkermansia*, and *norank_f_Muribaculaceae* and Iba1, and positive correlations between *Escherichia-Shigella*, *Enterococcus*, *Parabacteroides*, *Tyzzerella_4*, and *unclassified_f_Enterobacteriaceae* and Iba1, indicating that gut microbiota could modulate neuroinflammation of HFD offspring (Figure 6F).

## Discussion

The development of neurological reflexes is an efficacious and reliable indicator of the neurodevelopment of pups. Different reflex behaviors during development can reflect the maturity of the nervous system and physical development. For example, righting reflex measures the development of muscle and motor function, cliff avoidance reflects the sensory-motor function, and negative geotaxis evaluates the maturation of cranio-caudal coordination (29, 30). Nutritional status in early life is a major determinant of neurodevelopment. Maternal HFD during pregnancy and lactation adversely affect the neurodevelopment of offspring, as evidenced by delayed maturation of physiological reflexes (31). Dietary supplementation of velvet antler in dams improved the acquisition of righting reflex, cliff avoidance and negative geotaxis in offspring (32). Supplementation with MFGM in infant formula-fed pups narrowed the gap in the maturation age of cliff avoidance and negative geotaxis compared with breast-fed pups (33). Consistent with these findings, the present study proved that exposure to maternal HFD delayed the maturation of righting reflex and cliff avoidance, while maternal MFGM administration during pregnancy and lactation could restore the development of neurological reflexes in the offspring, suggesting that MFGM is beneficial to neurodevelopment.

Neurogenesis in the hippocampus, including cell proliferation and cell survival, is critical for neurodevelopment. New neurons in the subgranular layer of the DG continuously generate, maturate, and integrate functionally into existing neural circuits, thereby promoting neurodevelopment and behavioral reflexes. Maternal nutrition affects hippocampal neurogenesis in offspring during early development. Maternal HFD impaired neurogenesis during offspring hippocampal development (6). Maternal choline supplementation partially normalized neurogenesis in the offspring with Down syndrome (34). In the present study, maternal MFGM supplementation restored maternal HFD-induced reduction in the neurogenesis of weaned offspring, as evidenced by elevation of Ki-67 and DCX positive neurons, contributing to improved neurodevelopment. MFGM contains abundant polar lipids including phosphatidylcholine (PC), phosphatidylethanolamine (PE), sphingomyelin (SM), and gangliosides. Polar lipids and their metabolites such as long-chain unsaturated fatty acids and choline can be transmitted to offspring through placenta and breast milk and promote neurodevelopment (35–37). Maternal LC-PUFAs and choline supplementation were related to better memory and intelligence quotient in children (38, 39). Maternal supplementation with complex milk lipid during pregnancy and lactation increased ganglioside level in the brain of offspring, which was important for neurogenesis (40). Therefore, abundant polar lipids in MFGM contributed a lot to the beneficial effects of MFGM on neurodevelopment.

The mechanism of neurodevelopment is complex and can be regulated by multiple pathways. In recent years, communications between gut microbiota and brain have attracted a lot of attention. Based on the role of gut microbiota in neurogenesis and microglia maturation demonstrated in germ-free animals, disturbances of gut microbiota during development may affect neurodevelopment (14). Human studies showed that the composition of gut microbiota in infants was related to the diet and weight of mothers during pregnancy, and the β-diversity and α-diversity of gut microbiota in children were altered by maternal obesity (15). Meanwhile, the concentrations of pathogenic bacteria were elevated in infants of overweight mothers (16), which were associated with the occurrence of necrotizing enterocolitis in infants (41). Therefore, maternal obesity may have a negative impact on early immune development. *Akkermansia*, novel beneficial bacteria, was higher in lean children compared with overweight/obese children. *Lactobacillus* abundance is positively correlated with memory (42), and supplementation of omega-3 fatty acids in pregnant mice increased fecal levels of *Lactobacillus*, thereby enhancing social and cognitive function (43). *Escherichia shigella* was a leading cause of diarrhea in children and was inversely associated with cognitive scores (44). Preterm newborns with gut microbiota dominated by *Enterococcus* were significantly associated with death after 4 weeks of age or the neurodevelopment at 2 years of age (45). In this study, the overall structure of gut microbiota in offspring was changed due to maternal HFD. The abundance of *Escherichia shigella* and *Enterococcus* were up-regulated, and *Akkermansia* was down-regulated in the weaned offspring of obese dams. However, maternal MFGM intervention could significantly reverse these changes. Of note, there was no significant change in the *Lactobacillus* abundance of HFD offspring, which was increased by MFGM intervention in both control and obese dams in the present study. Therefore, the modulation of MFGM on gut microbiota might promote the development of immune and cognition in offspring.

Maternal diet could affect the gut microbiota of offspring through vertically transmitted to the offspring during delivery, thus diet-induced changes in the gut microbiota of the mothers can directly affect the colonization of the gut microbiota of offspring (46). A previous study has found that administration of polar lipids-enriched MFGM in obese dams during pregnancy and lactation could restore the ratio of *Firmicutes* to *Bacteroides*, reduce the relative abundance of *Ruminococcaceae* and *Enterococcus*, and increase the relative abundance of *Akkermansia* (25), which were similar to the changes in the gut microbiota of offspring observed in the present study. Therefore, the beneficial effect of MFGM on the gut microbiota of offspring may be attributed to the improvement of the gut microbiota of dams. The benefits of MFGM in regulating gut microbiota may be attributed to its components and their metabolites. Dietary milk SM altered gut microbiota composition in HFD mice, with significantly reduced relative abundance of Gram-negative phyla, such as *Bacteroidetes* and *Tenericutes*, and the major digestion products of SM, sphingosine, exhibited strong antimicrobial properties against pathogenic bacteria *in vitro* (47, 48). Ethanolamine, which is the base constituent of PE, was found to be helpful for the development of infant intestine by improving intestinal antioxidant capacity, promoting intestinal cell differentiation and altering gut microbiota (49). Gangliosides reduced the relative content of *Escherichia coli* in preterm newborn infants and increased fecal *Bifidobacteria* counts (50). Therefore, the regulatory effect of MFGM on the gut microbiota of offspring may be due to simultaneous effects on both dams and offspring.

Microbiota shifts are well known to be associated with inflammation. LPS from the cell wall of Gram-negative bacteria in the gut microbiota is an important cause of systemic low-grade inflammation. LPS can activate the immune system through toll-like receptor 4 and downstream inflammatory signaling molecules, thereby promoting the production of inflammatory factors including IL-1β, IL-6, TNF-α (51). Exposure to maternal chronic low-grade inflammation induced by long-term HFD during pregnancy and lactation could also negatively affect the serum cytokine levels of offspring (52). In this study, offspring of obese dams had high levels of serum LPS and inflammatory factors at weaning, while MFGM intervention suppressed the inflammatory state of the offspring. Gut microbiome profile was linked to the inflammatory state of the host. For example, *Lactobacillus* could repair the intestinal mucosal barrier and prevent LPS and harmful bacteria from entering the circulation through the intestinal epithelium (53). *Muribaculaceae*, which produce butyrate, is associated with the degradation of complex carbohydrates, the formation of mucus layer in the colon and the improvement of barrier function (54). Moutan cortex polysaccharides up-regulated the abundance of *Lactobacillus* and *Muribaculaceae_unclassified*, thus improving intestinal barrier and inflammatory response in diabetic rats (55). In addition, overweight during pregnancy reduced the abundance of *Blautia* in the gut microbiota of newborns (56), which was associated with the deterioration of intestinal inflammation and metabolic phenotype in obese children (57). Except for the reduction in anti-inflammatory bacteria, the increase in pro-inflammatory bacteria also contributes to the inflammation. *Enterococcus* in infants fed with infant formula was significantly higher than that in breast-fed infants, which was a leading cause of sepsis in infants (58). Through correlation analysis in the present study, we found that *Lactobacillus*, *norank_f_Muribaculaceae*, *Akkermansia*, *Blautia*, *Escherichia-Shigella*, and *Enterococcus* were significantly correlated with the change of inflammation in offspring, demonstrating that maternal MFGM supplementation improved the inflammation of offspring *via* regulating gut microbiota.

Cytokines in circulation induced by LPS could damage the blood-brain barrier *via* binding to the endothelial receptors and releasing pro-inflammatory mediators (59). Subsequently, LPS and pro-inflammatory cytokines enter the brain, inhibit the phagocytosis of microglia and stimulate the production of pro-inflammatory cytokines, thereby contributing to microglia activation and the occurrence of neuroinflammation (60). In the present study, since the changes in inflammation-related bacteria and the improvement of gut microbiota in systemic inflammation have been verified, the effect of MFGM on neuroinflammation was further analyzed. Rats fed HFD from 4 weeks before mating to the end of lactation increased the expression of hippocampal microglial activation marker Iba1 in offspring at birth, and hippocampal IL-1β levels at weaning and adulthood were significantly higher than those in the control offspring (61). Consistent with this study, in our study, elevated inflammatory mediators and microglial activation were observed in HFD offspring, which were alleviated by maternal MFGM supplementation. According to previous reports, gut microbiota could regulate the maturation and immune response of microglia through metabolites such as short-chain fatty acids, thus modulating neurodevelopment (62). Meanwhile, changes in some bacteria have been proved to be associated with neuroinflammation and cognition. *Lactobacillus plantarum* supplementation improved memory impairment in Alzheimer’s disease mice *via* decreasing *Enterobacter* abundance and increasing *Lactobacillus* and *Bifidobacterium* abundance, reducing LPS levels in blood and feces, and inhibiting microglial activation (63). Early life HFD could damage neurodevelopment of mice and significantly reduce the abundance of *Akkermansia*, while supplementation of *Akkermansia* significantly reduced the activation of microglia and the expression of pro-inflammatory cytokines, thereby improving learning and memory ability (64). Increased *Escherichia Shigella* abundance was associated with peripheral inflammatory states in patients with cognitive impairment and brain amyloidosis (65). In the present study, the correlation between gut microbiota and neuroinflammation was further confirmed by Spearman’s correlation analysis, and bacteria including *Lactobacillus*, *Akkermansia*, *Escherichia-Shigella*, and *Enterococcus* were significantly correlated with the change of microglial activation in offspring, suggesting that MFGM could improve neuroinflammation at least partially *via* modulating gut microbiota.

## Conclusion

**FIGURE 7:**
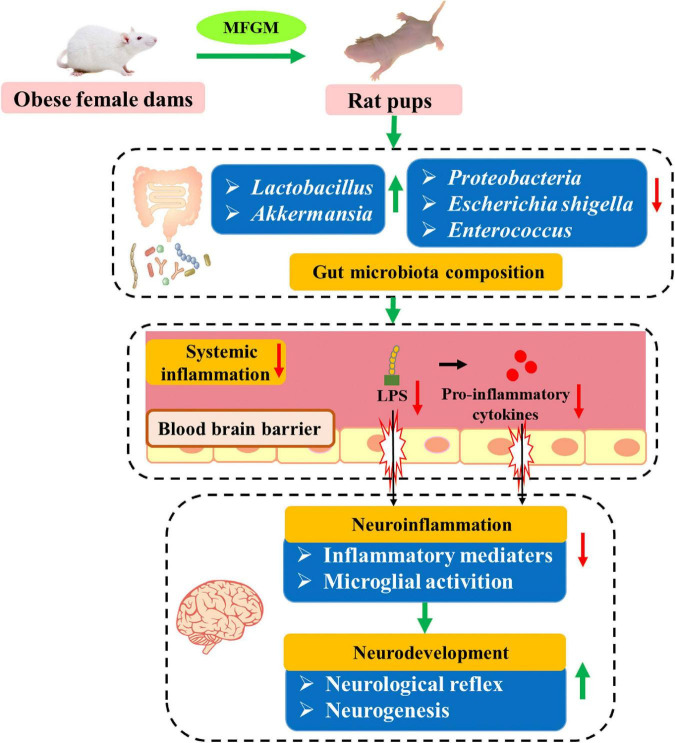
Possible mechanism for the beneficial effect of maternal MFGM supplementation on the neurodevelopment of offspring.

In summary, supplementation of MFGM to HFD-induced obese dams during pregnancy and lactation reduced postnatal body weight of offspring, promoted the maturation of neurological reflexes and hippocampal neurogenesis in the offspring. MFGM modulated the diversity of gut microbiota, downregulating the abundance of pro-inflammatory bacteria such as *Escherichia shigella* and *Enterococcus*, and upregulating the abundance of bacteria with anti-inflammatory and anti-obesity functions, such as *Akkermansia* and *Lactobacillus*. MFGM also reduced the levels of LPS and pro-inflammatory cytokines (IL-1β, IL-6, TNF-α) in the serum and brain tissue of the offspring, and inhibited the expression of microglial activation marker Iba1, which were beneficial to reducing the neuroinflammation of offspring. The correlation between the changes in the gut microbiota and inflammation was further verified. Therefore, gut microbiota-mediated reduction of inflammatory response was the potential mechanism by which MFGM stimulated neurodevelopment (Figure 7). These findings provide new evidence of the MFGM as an effective functional component for neurodevelopment in early life.

## Data availability statement

The raw data of 16S rRNA gene sequence analysis can be found online at: https://www.ncbi.nlm.nih.gov/sra/PRJNA847149.

## Ethics statement

The animal study was reviewed and approved by the Ethics Committee of China Agricultural University.

## Author contributions

QY, HG, and XM contributed to the conception and design of the study. QY and HG conducted experiments and analyzed the data. QY wrote the manuscript. MD, TL, and XM revised the manuscript. All authors contributed to the article and approved the submitted version.
