# Supplementary material for: Milk fat globule membrane supplementation to obese rats during pregnancy and lactation promotes neurodevelopment in offspring via modulating gut microbiota
Source: Front Nutr. 2022 Aug 15;9:945052. doi: 10.3389/fnut.2022.945052 (PMC9421050; doi:10.3389/fnut.2022.945052)
Supplement: Supplementary file 1 [file Data_Sheet_1.docx]

Supplementary Material

# Supplementary Figures and Tables

## Supplementary Figures


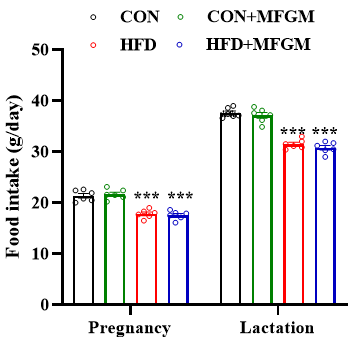


**Supplementary Figure 1.** Food intake of dams during pregnancy and lactation

## Supplementary Tables

**Supplementary Table 1.** Caloric information of different diets (provided by Research Diets).

| Caloric Information | D12450J | D12492 | D12451 |
| --- | --- | --- | --- |
| Protein (% Kcal) | 20 | 20 | 20 |
| Fat (% Kcal) | 10 | 60 | 45 |
| Carbohydrate (% Kcal) | 70 | 20 | 35 |
| Energy density (Kcal/g) | 3.82 | 5.21 | 4.7 |
